# Clinical outcomes of simultaneous phototherapeutic keratectomy and photoastigmatic keratectomy

**DOI:** 10.1038/s41598-021-89044-3

**Published:** 2021-05-04

**Authors:** Kazutaka Kamiya, Kana Yazaki, Wakako Ando, Masahide Takahashi, Nobuyuki Shoji

**Affiliations:** 1 Visual Physiology, School of Allied Health Sciences, Kitasato University, 1-15-1 Kitasato, Minami, Sagamihara, Kanagawa 252-0373 Japan; 2 Department of Ophthalmology, School of Medicine, Kitasato University, Sagamihara, Japan

**Keywords:** Eye diseases, Outcomes research

## Abstract

This study was aimed to assess the outcomes of simultaneous phototherapeutic keratectomy (PTK) and photoastigmatic keratectomy (PAK), with special attention to astigmatic correction. We comprised 70 eyes of 70 patients who underwent simultaneous PTK and PAK in patients having granular corneal dystrophy and band keratopathy with refractive astigmatism of 1 diopter (D) or more. Preoperatively and 6 months postoperatively, we assessed corrected uncorrected distance visual acuity (UDVA), distance visual acuity (CDVA), manifest spherical equivalent, refractive astigmatism, corneal astigmatism, and higher-order aberrations (HOAs). LogMAR CDVA significantly improved, from 0.27 ± 0.27 preoperatively, to 0.13 ± 0.21 postoperatively (Paired t test, p < 0.001). LogMAR UDVA also significantly improved, from 0.70 ± 0.32 preoperatively, to 0.57 ± 0.41 postoperatively (p = 0.043). Refractive astigmatism significantly decreased, from 2.12 ± 0.95 D preoperatively, to 0.89 ± 0.81 D postoperatively (p < 0.001). Corneal astigmatism also significantly decreased, from 2.17 ± 0.90 D preoperatively, to 1.08 ± 0.71 D postoperatively (p < 0.001). Corneal HOAs did not significantly change, from 0.54 ± 0.30 µm preoperatively, to 0.48 ± 0.20 µm postoperatively (p = 0.140). No significant complications occurred in any eye. Simultaneous PTK and PAK treatment is effective not only for improving visual acuity, but also for reducing astigmatism.

## Introduction

Phototherapeutic keratectomy (PTK) has been widely accepted as an effective treatment for corneal diseases such as granular corneal dystrophy (GCD) and band keratopathy (BK) in order to reduce the diseased opacity and to increase the transparency of the cornea using excimer laser photoablation^1–3^. It is well known that PTK induces a hyperopic shift in refraction, but that there are not many concerns about astigmatic correction in such patients having opaque cornea. Actually, some of the patients had a large amount of astigmatism after PTK. Astigmatic correction plays a vital role for such patients having a diseased cornea, in order to maximize visual performance and subsequent patient satisfaction even after PTK. Considering that both PTK and photoastigmatic keratectomy (PAK) require the same excimer laser photoablation, we postulate that simultaneous PTK and PAK may be beneficial for GCD and BK with some astigmatism. As far as we can ascertain, this is the first case series to evaluate the outcomes of simultaneous PTK and PAK in such patients, with special attention to astigmatic correction.

## Results

### Study population

The preoperative demographics of the study population is listed in Table 1. The patient age at the time of surgery was 69.1 ± 11.6 years. All surgeries were uneventful, and no intraoperative complications were observed. No eyes were lost during the 6-month follow-up in this series.

**Table 1:** Preoperative and postoperative demographics of the study population undergoing simultaneous phototherapeutic keratectomy and photoastigmatic keratectomy.

Total (N = 70)	Preoperative	Postoperative (6-months)	P value
Age (years)	69.1 ± 11.6 years (95% CI, 46.5 to 91.8 years)		
Gender (male:female)	30:40		
Manifest spherical equivalent	− 1.56 ± 2.91 D (95% CI, − 7.27 to 4.15 D)	1.71 ± 2.19 D (95% CI, − 2.59 to 6.01 D)	< 0.001
Refractive astigmatism	2.12 ± 0.95 D (95% CI, 0.26 to 3.98 D)	0.89 ± 0.81 D (95% CI, − 0.70 to 2.49 D)	< 0.001
Corneal astigmatism	2.17 ± 0.90 D (95% CI, 0.41 to 3.93 D)	1.08 ± 0.71 D (95% CI, − 0.32 to 2.48 D)	< 0.001
LogMAR UDVA	0.70 ± 0.32 (95% CI, 0.08 to 1.32)	0.57 ± 0.41 (95% CI, − 0.23 to 1.38)	0.043
LogMAR CDVA	0.27 ± 0.27 (95% CI, − 0.26 to 0.80)	0.13 ± 0.21 (95% CI, − 0.28 to 0.55)	< 0.001
Corneal HOAs	0.54 ± 0.30 µm (95% CI, − 0.05 to 1.14 µm)	0.48 ± 0.20 µm (95% CI, 0.08 to 0.88 µm)	0.140
Granular corneal dystrophy (N = 34)	Preoperative	Postoperative (6-months)	P value
Age (years)	62.5 ± 10.0 years (95% CI, 43.0 to 82.0 years)		
Gender (male:female)	10:24		
Manifest spherical equivalent	− 2.08 ± 2.49 D (95% CI, − 6.96 to 2.80 D)	1.99 ± 2.20 D (95% CI, − 2.31 to 6.30 D)	< 0.001
Refractive astigmatism	2.04 ± 0.79 D (95% CI, 0.50 to 3.59 D)	0.88 ± 0.81 D (95% CI, − 0.70 to 2.47 D)	< 0.001
Corneal astigmatism	2.32 ± 0.96 D (95% CI, 0.44 to 4.20 D)	1.08 ± 0.73 D (95% CI, − 0.36 to 2.52 D)	< 0.001
LogMAR UDVA	0.74 ± 0.27 (95% CI, 0.22 to 1.27)	0.55 ± 0.38 (95% CI, − 0.21 to 1.30)	0.035
LogMAR CDVA	0.30 ± 0.22 (95% CI, − 0.13 to 0.74)	0.11 ± 0.18 (95% CI, − 0.23 to 0.46)	< 0.001
Corneal HOAs	0.51 ± 0.30 µm (95% CI, − 0.07 to 1.09 µm)	0.50 ± 0.20 µm (95% CI, 0.11 to 0.88 µm)	0.553
Band keratopathy (N = 36)	Preoperative	Postoperative (6-months)	P value
Age (years)	75.4 ± 9.3 years (95% CI, 57.2 to 93.7 years)		
Gender (male:female)	20:16		
Manifest spherical equivalent	− 1.07 ± 3.22 D (95% CI, − 7.38 to 5.24 D)	1.45 ± 2.19 D (95% CI, − 2.84 to 5.74 D)	< 0.001
Refractive astigmatism	2.19 ± 1.09 D (95% CI, 0.07 to 4.32 D)	0.80 ± 0.83 D (95% CI, − 0.73 to 2.54 D)	< 0.001
Corneal astigmatism	2.04 ± 0.83 D (95% CI, 0.41 to 3.66 D)	1.08 ± 0.70 D (95% CI, − 0.30 to 2.46 D)	< 0.001
LogMAR UDVA	0.65 ± 0.35 (95% CI, − 0.04 to 1.35)	0.60 ± 0.44 (95% CI, − 0.26 to 1.47)	0.515
LogMAR CDVA	0.23 ± 0.31 (95% CI, − 0.37 to 0.84)	0.15 ± 0.24 (95% CI, − 0.32 to 0.63)	0.034
Corneal HOAs	0.58 ± 0.31 µm (95% CI, − 0.03 to 1.18 µm)	0.46 ± 0.21 µm (95% CI, 0.05 to 0.87 µm)	0.111

D = diopter, LogMAR = logarithm of the minimal angle of resolution, UDVA = uncorrected distance visual acuity, CDVA = corrected distance visual acuity, HOAs = higher-order aberrations.

### Visual and refractive outcomes

Logarithm of the minimum angle of resolution (logMAR) corrected distance visual acuity (CDVA) significantly improved from 0.27 ± 0.27 preoperatively to 0.13 ± 0.21 postoperatively (Paired t test, p < 0.001). Uncorrected distance visual acuity (UDVA) also significantly improved from 0.70 ± 0.32 preoperatively to 0.57 ± 0.41 postoperatively (p = 0.043). Mean manifest refraction was significantly hyperopic from − 1.56 ± 2.91 diopter (D) preoperatively to 1.71 ± 2.19 D postoperatively (p < 0.001).

### Astigmatic outcomes

Refractive astigmatism significantly decreased from 2.12 ± 0.95 D preoperatively to 0.89 ± 0.81 D postoperatively (p < 0.001). Corneal astigmatism also significantly decreased from 2.12 ± 0.95 D preoperatively to 1.08 ± 0.71 D postoperatively (p < 0.001).

**Figure 1:**
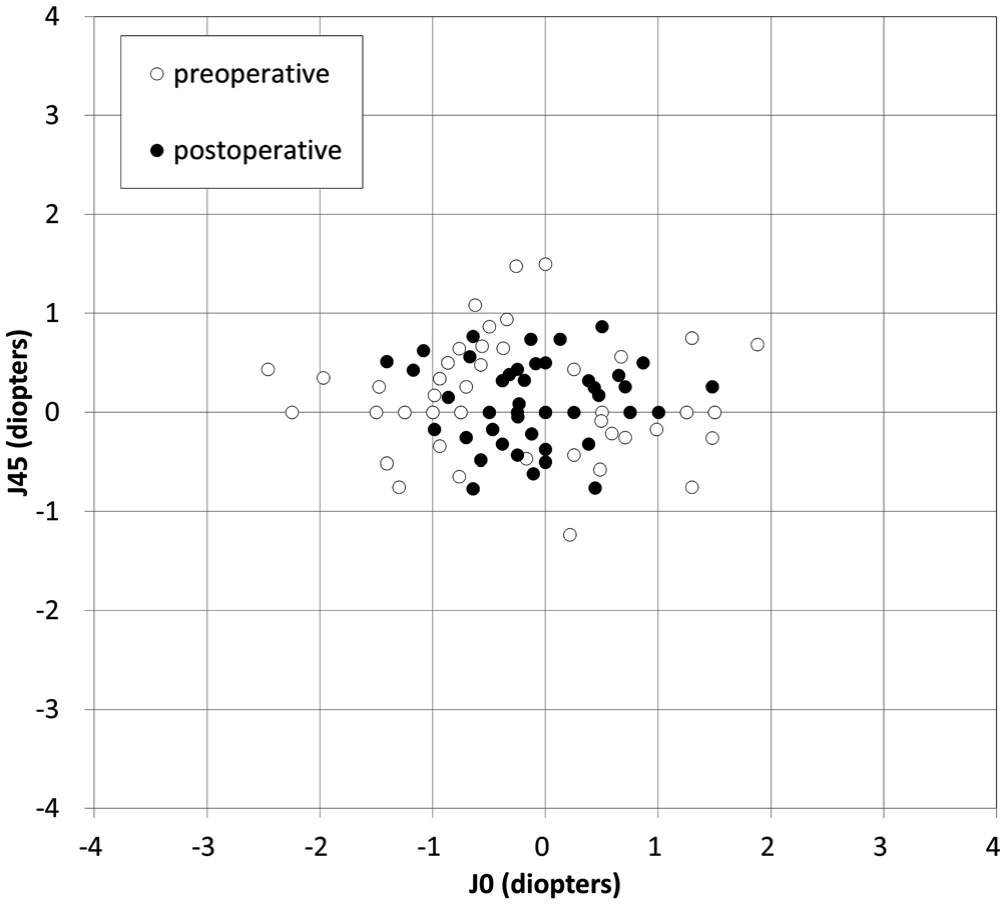
Power vector analysis of refractive astigmatism before and after simultaneous phototherapeutic keratectomy and photoastigmatic keratectomy.

**Figure 2:**
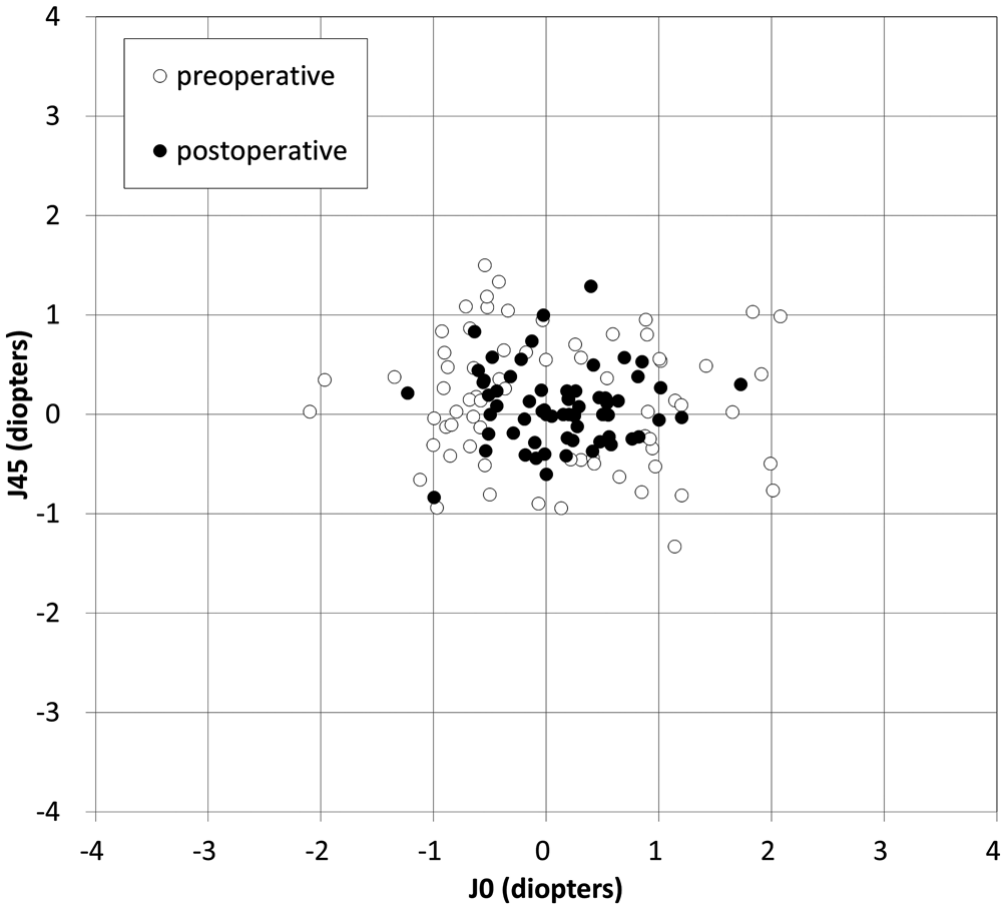
Power vector analysis of corneal astigmatism before and after simultaneous phototherapeutic keratectomy and photoastigmatic keratectomy.

Power vector analyses of refractive and corneal astigmatism are summarized in Table 2. For refractive and corneal astigmatism, the dispersed cluster of points preoperatively tended to collapse around the origin postoperatively, indicating a reduction in vector astigmatic change (Figs. 1 and 2). For refractive astigmatism, 77% and 94% of eyes were within ± 0.5 and 1.0 D, respectively, for J_0_, and 86% and 100% of eyes were within ± 0.5 and 1.0 D, respectively, for J_45_. For corneal astigmatism, 66% and 94% of eyes were within ± 0.5 and 1.0 D, respectively, for J_0_, and 86% and 99% of eyes were within ± 0.5 and 1.0 D, respectively, for J_45_.

**Table 2:** Preoperative and postoperative refractive astigmatism, corneal astigmatism and refraction after vectorial conversion in eyes undergoing simultaneous phototherapeutic keratectomy and photoastigmatic keratectomy.

	Preoperative	Postoperative (6-months)	P value
**Refractive astigmatism**			
Amount (D)	2.12 ± 0.95 D (95% CI, 0.26 to 3.98 D)	0.89 ± 0.81 D (95% CI, − 0.70 to 2.49 D)	< 0.001
M	− 1.56 ± 2.91 D (95% CI, − 7.27 to 4.15 D)	1.71 ± 2.19 D (95% CI, − 2.59 to 6.01 D)	< 0.001
J_0_	− 0.25 ± 1.01 D (95% CI, − 2.23 to 1.72 D)	− 0.09 ± 0.49 D (95% CI, − 1.04 to 0.87 D)	0.109
J_45_	0.09 ± 0.53 D (95% CI, − 0.95 to 1.12 D)	0.08 ± 0.34 D (95% CI, − 0.59 to 0.74 D)	0.902
B	2.78 ± 2.11 D (95% CI, − 1.36 to 6.93 D)	2.25 ± 1.74 D (95% CI, − 1.16 to 5.66 D)	0.138
**Corneal astigmatism**			
Amount (D)	2.17 ± 0.90 D (95% CI, 0.41 to 3.93 D)	1.08 ± 0.71 D (95% CI, − 0.32 to 2.48 D)	< 0.001
J_0_	0.06 ± 0.98 D (95% CI, − 1.87 to 1.98 D)	0.13 ± 0.52 D (95% CI, − 0.89 to 1.15 D)	0.406
J_45_	0.14 ± 0.64 D (95% CI, − 1.12 to 1.40 D)	0.07 ± 0.36 D (95% CI, − 0.64 to 0.78 D)	0.380

CI = confidence interval, D = diopter, M = spherical equivalent refraction, J_0_ = Jackson cross-cylinder, axes at 0 and 90 degrees, J_45_ = Jackson cross-cylinder, axes at 45 and 135 degrees, B = blur strength.

### Corneal higher-order aberrations

Corneal higher-order aberrations (HOAs) did not significantly change from 0.54 ± 0.30 µm preoperatively to 0.48 ± 0.20 µm postoperatively (p = 0.140).

### Adverse events

We found transient haze formation in 5 eyes (7%) 1 month postoperatively, but all these eyes recovered with time. Otherwise, we found no postoperative complications such as keratectasia, central island formation, or infectious keratitis, during the observation period.

## Discussion

In the current study, our results showed that simultaneous PTK and PAK significantly improved CDVA, and that it significantly decreased both corneal and refractive astigmatism. It is suggested that simultaneous PTK and PAK is a viable option not only for improving visual performance, but also for reducing astigmatism, in eyes requiring PTK with some amount of astigmatism. To our knowledge, this is the first case series on the outcomes of simultaneous PTK and PAK in such patients. We believe that this combined surgical technique will be beneficial for maximizing visual quality and subsequent patient satisfaction after PTK in daily practice, because we can easily and quickly perform this combined technique using the same excimer laser system. Although approximately 0.89 D of astigmatism still remained after surgery, we assume that this novel approach is clinically acceptable, because it is still challenging to measure corneal and refractive astigmatism for such diseased patients.

It has been reported that conventional PTK usually induced a hyperopic shift in refraction by approximately 2.0 to 3.0 D^4–7^, which is comparable with our current findings. In our study, UDVA significantly improved after simultaneous PTK and PAK, possibly because the preoperative mean refraction was slightly myopic (− 1.56 ± 2.91 D) in the study population, in addition to the reduction of astigmatism. We should be aware that this hyperopic shift can result in a decrease in UDVA, especially when the preoperative refraction is emmetropia to hyperopia. A topical ethylene-diamine-tetra-acetic acid (EDTA) chelation has been widely applied for eyes with BK, as a cost-effective treatment, since it does not require an excimer laser system. Although this treatment did not induce a significant change in refraction, Al-Hity recently demonstrated, in a large study of 89 eyes, that there was no evidence of a significant difference between the initial and final visual acuity following EDTA treatment^8^. We also assume that PTK has advantages over EDTA chelation in terms of visual recovery, because the former treatment can provide a smoother corneal surface than the latter treatment.

There are at least two limitations to this study. One is that the follow-up time is rather limited, since the corneal shape usually stabilized at 6 months postoperatively, in consideration of the general wound-healing and biomechanical responses of the cornea induced by excimer laser photoablation. A further long-term observation would be ideal to confirm the authenticity of our findings. Another limitation is that it is still difficult to exactly measure the magnitude and the axis of corneal and refractive astigmatism as well as corneal HOAs, in such opaque eyes having advanced GCD or BK in a clinical setting. We confirmed that reliable and reproducible corneal measurements were obtained in all patients by experienced optometrists before surgery.

In summary, our results may support the view that simultaneous PTK and PAK treatment is effective not only for improving visual performance, but also for reducing astigmatism, without any significant postoperative complications. Considering that this combined surgical technique can be simultaneously and easily applied using the same excimer laser system, we believe that it may hold a promise as an effective means for improving visual acuity and reducing astigmatism instantaneously.

## Methods

### Study population

We registered the study protocol with the University Hospital Medical Information Network Clinical Trial Registry (000040137)(13/04/2020). Seventy eyes of 70 consecutive patients (30 male and 40 female) who underwent simultaneous PTK and PAK for the treatment of GCD and BK with refractive astigmatism of 1 D or more, at Kitasato University Hospital, were included in this case series. Advanced cases of GCD and BK in whom we could not obtain reliable and reproducible corneal measurements, were excluded from this study. The sample size in this study offered 99.9% statistical power at the 5% level in order to detect a 1-D difference in astigmatism, when the standard deviation (SD) of the mean difference was 1.6 D. Preoperatively and 6 months postoperatively, we evaluated CDVA, UDVA, manifest spherical equivalent, refractive astigmatism (manifest cylinder), corneal astigmatism, and corneal HOAs. Corneal astigmatism on the central 15° ring (equal to the 3.0-mm ring) and corneal HOAs for a 4-mm pupil were automatically measured with a Scheimpflug anterior segment photography system (Pentacam HR, Oculus, Wetzlar, Germany). After achieving perfect alignment, we took 25 Scheimpflug images within 2 s with this instrument. We checked the image quality for each eye, and only one examination with a high-quality factor was recorded. A retrospective data review was approved by the Institutional Review Board of Kitasato University (B20-065) and followed the tenets of the Declaration of Helsinki. Written informed consent was obtained from all patients for this surgery.

### Power vector analysis

We converted manifest refraction to the power vector coordinates (M: spherical equivalent, J_0_, J_45_, Jackson crossed cylinders), as described previously^9^, and B: overall blurring strength, using the following formulas:

$$M = S + C/2,J_{0} = \left( { - C/2} \right)\cos \left( {2\alpha } \right),J_{45} = \left( { - C/2} \right)\sin \left( {2\alpha } \right),B = \left( {M^{2} + J_{0}^{2} + J_{45}^{2} } \right)^{1/2} ,$$

where M is the spherical equivalent; S is the sphere; C is the cylinder; J_0_ is the Jackson cross-cylinder, axes at 0 and 90 degrees; α is the axis; J_45_ is the Jackson cross-cylinder, axes at 45 and 135 degrees; and B is the overall blurring strength of the refractive error.

### Surgical procedures

Simultaneous PTK and PAK was performed using the VISX STAR S4 IR excimer laser system (Johnson & Johnson Vision, Santa Ana, USA) with the following parameters: wavelength, 193 nm; fluency, 160 mJ/cm^2^; repetition rate, 10 Hz; ablation zone diameter, 6.0 mm; transition zone, 0.5 mm; and ablation depth, 39.7 ± 8.9 µm (range, 21 to 59 μm). The ablation depth was determined based on the clinical appearance of the depth of pathology using slit-lamp microscopy, and the diopter was inversely calculated by the ablation depth. We basically selected the ablation depth and the refractive correction as 44 to 46 µm and 3.5 D in eyes with GCD, and as 32 to 34 µm and 2.5 D in eyes with BK, respectively, dependent on the keratometry. We used the transepithelial technique to remove the corneal epithelium (in depth of 50 µm). In order to reduce the possible risk of central island (CI) formation, we applied the photorefractive keratectomy (PRK) mode (with anti-CI program), instead of the PTK mode (without anti-CI program), based on our findings that the use of the PRK mode significantly decreased the rate of CI formation, and subsequently improved corrected visual acuity, compared to that of the PTK mode^10,11^.

After PTK surgery, PAK was subsequently performed in order to fully correct refractive astigmatism. The estimated total postoperative corneal thickness was at least 400 µm or more to prevent the occurrence of iatrogenic keratectasia. Eyes with keratoconus were excluded from the study by using the keratoconus screening test of Placido disk videokeratography (TMS-4, Tomey, Nagoya, Japan).

Postoperatively, steroidal (0.1% fluorometholone) and antibiotic (1.5% levofloxacin) medications were topically administered 4 times daily for 1 week, after insertion of a soft contact lens, and the dose was steadily reduced thereafter.

### Statistical analysis

The normality of data was firstly checked using the Kolmogorov–Smirnov test. Because we confirmed that the data were normally distributed, the paired t test was utilized to compare the pre- and post-treatment. The results are expressed as the mean ± SD, and a value of p less than 0.05 was deemed statistically significant.

### Ethics approval

The study was approved by the Institutional Review Board of Kitasato University (B20-065) and followed the tenets of the Declaration of Helsinki.
